# SARS-Cov-2 infection and neuropathological findings: a report of 18 cases and review of the literature

**DOI:** 10.1186/s40478-023-01566-1

**Published:** 2023-05-10

**Authors:** Laetitia Lebrun, Lara Absil, Myriam Remmelink, Ricardo De Mendonça, Nicky D’Haene, Nicolas Gaspard, Stefan Rusu, Marie-Lucie Racu, Amandine Collin, Justine Allard, Egor Zindy, Andrea Alex Schiavo, Sarah De Clercq, Olivier De Witte, Christine Decaestecker, Maria-Beatriz Lopes, Isabelle Salmon

**Affiliations:** 1 Department of Pathology, Erasme University Hospital, Université Libre de Bruxelles (ULB)Hôpital Universitaire de Bruxelles (HUB), CUB Hôpital Erasme, 808 Route de Lennik, B-1070 Brussels, Belgium; 2 Department of Neurology, Université Libre de Bruxelles (ULB), Hôpital Universitaire de Bruxelles (HUB), CUB Hôpital Erasme, Erasme University Hospital, Brussels, Belgium; 3 DIAPath, Center for Microscopy and Molecular Imaging (CMMI), ULB, Gosselies, Belgium; 4 Department of Neurosurgery, Université Libre de Bruxelles (ULB)Hôpital Universitaire de Bruxelles (HUB), CUB Hôpital ErasmeErasme University Hospital, Brussels, Belgium; 5 Laboratory of Image Synthesis and Analysis, Brussels School of Engineering/École Polytechnique de Brussels, ULB, Brussels, Belgium; 6 Department of Pathology, University of Virginia Health System, Charlottesville, VA USA

**Keywords:** SARS-CoV-2, COVID-19, PCR, Postmortem procedure, Immunohistochemistry

## Abstract

**Introduction:**

COVID-19-infected patients harbour neurological symptoms such as stroke and anosmia, leading to the hypothesis that there is direct invasion of the central nervous system (CNS) by SARS-CoV-2. Several studies have reported the neuropathological examination of brain samples from patients who died from COVID-19. However, there is still sparse evidence of virus replication in the human brain, suggesting that neurologic symptoms could be related to mechanisms other than CNS infection by the virus. Our objective was to provide an extensive review of the literature on the neuropathological findings of postmortem brain samples from patients who died from COVID-19 and to report our own experience with 18 postmortem brain samples.

**Material and methods:**

We used microscopic examination, immunohistochemistry (using two different antibodies) and PCR-based techniques to describe the neuropathological findings and the presence of SARS-CoV-2 virus in postmortem brain samples. For comparison, similar techniques (IHC and PCR) were applied to the lung tissue samples for each patient from our cohort. The systematic literature review was conducted from the beginning of the pandemic in 2019 until June 1st, 2022.

**Results:**

In our cohort, the most common neuropathological findings were perivascular haemosiderin-laden macrophages and hypoxic-ischaemic changes in neurons, which were found in all cases (n = 18). Only one brain tissue sample harboured SARS-CoV-2 viral spike and nucleocapsid protein expression, while all brain cases harboured SARS-CoV-2 RNA positivity by PCR. A colocalization immunohistochemistry study revealed that SARS-CoV-2 antigens could be located in brain perivascular macrophages.

The literature review highlighted that the most frequent neuropathological findings were ischaemic and haemorrhagic lesions, including hypoxic/ischaemic alterations. However, few studies have confirmed the presence of SARS-CoV-2 antigens in brain tissue samples.

**Conclusion:**

This study highlighted the lack of specific neuropathological alterations in COVID-19-infected patients. There is still no evidence of neurotropism for SARS-CoV-2 in our cohort or in the literature.

**Supplementary Information:**

The online version contains supplementary material available at 10.1186/s40478-023-01566-1.

## Introduction

Human coronaviruses are responsible for multiple respiratory diseases of varying severity and, in some cases, are associated with rapid evolution. The first COVID outbreak of severe acute respiratory syndrome coronavirus (SARS-CoV) began in 2002 (SARS-CoV-1) with 10% mortality, and one decade later, Middle East respiratory syndrome coronavirus (MERS-CoV) was the second COVID outbreak with 35% global fatality. Seven years later, in 2019, the SARS-CoV-2 pandemic started [1]. Coronavirus disease 2019 (COVID-19) caused by severe acute respiratory syndrome coronavirus 2 (SARS-CoV-2) has been associated with a high mortality rate, mostly due to severe pulmonary lesions [2, 3]. However, this disease is not limited to the lungs; broad and unspecific symptoms have been described. Indeed, COVID-19 can be seen as a systemic disease associated with, among others, cardiovascular and renal injuries [2] as well as gastrointestinal, hepatic or dermatologic complications [4]. Studies on the pathophysiology of this viral infection have proposed that SARS-CoV-2 can directly alter cell function by linking to the angiotensin converting enzyme 2 (ACE2) receptor, which is almost ubiquitous in the human body [2].

Neurological symptoms have also been described in COVID-19-infected patients, leading to some hypotheses that the central nervous system (CNS) compartment may be infected by the virus [5]. However, there is still sparse evidence of virus replication in the human brain suggesting that the neurological symptoms could be related to mechanisms other than direct CNS invasion by the virus. In the literature, neuropathological changes have been described in brain tissues of COVID-19-infected patients, especially neurovasculature injuries. The underlying mechanism could be related to complement activation leading to breakdown of the blood–brain barrier, microthromboses, perivascular inflammation and neuronal injury [6–9]. Nevertheless, few studies on a limited number of cases have been conducted on brain samples from patients who died from SARS-CoV-2 infection because of the rigorous guidelines for the biosafety control of autopsy practice. Some papers have reported the presence of SARS-CoV-2 antigens in the CNS by techniques such as PCR [10–12], but the presence of replicating virus in brain tissue and the mechanism of CNS infection have not yet been elucidated. The olfactory pathway is suggested as a portal of entry of SARS-CoV-2 in the brain. The involvement of isolated oculomotor, trochlear and facial nerves has been described [13]. Others suggested that the virus could infect the CNS compartment through infected dendritic or white blood cells [14].

In addition to comorbidities such as diabetes, obesity, and hypertension, COVID-19-infected patients also usually receive invasive treatments such as extracorporeal membrane oxygenation (ECMO) and mechanical ventilation (MV), which can cause neuropathological changes such as haemorrhage [15–17]. This can lead to bias and misunderstanding in the interpretation of the neuropathological findings in postmortem brain tissue studies.

**The aim of the study** was to provide an extensive review of the literature on the neuropathological findings found in COVID-19-infected patients and the different techniques used in the literature to highlight the presence of the virus in brain tissue. We also reported our own experience of neuropathological injuries as well as the distribution of the virus in 18 brain samples (and lung tissues for comparison) of patients who underwent autopsy in our institution.

## Materials and methods

### Study design

In this retrospective study, we included 18 adult patients (> 18 years) who died in our hospital (either in a COVID-19 unit or an intensive care unit) from March 13, 2020 to 21 June 2020 with confirmed SARS-CoV-2 infection (i.e., positive RT‒PCR assay (n = 16) and/or antigen test on nasopharyngeal swab and/or bronchoalveolar lavage specimen (n = 2)) and underwent autopsy with brain sampling. The exclusion criteria were lack of family consent for postmortem examination and a delay greater than 5 days between death and autopsy. The study protocol was approved by the local Ethics Committee (P2020/218).

### Clinical data

 Additional file 1: Table 1 details the clinical characteristics observed in our series. We collected relevant clinical data, including age, sex, comorbidities (hypertension, obesity, diabetes, cerebrovascular disease, chronic pulmonary disease, dyslipidaemia, cardiovascular disease), neurological history, neurological symptoms (if available), treatments (antivirals, antibiotics, anticoagulants, antiplatelet drugs, corticosteroids, ECMO and MV) and cause of death. The duration between death and autopsy was also calculated.

**Table 1:** Neuropathological findings in the postmortem brain samples of the COVID-19 patients in our series (n = 18)

N°	Inflammatory changes									Thrombotic/Arteriosclerosis changes									Ischemic/Haemorrhagic changes									Viral changes			Meninges	
	Oedema			Perivascular lymphocyte			Perivascular haemosiderin laden macrophages			Vasculitis			Arteriolosclerosis/Atherosclerosis			Thrombosis/Microthrombosis			Hypoxic-ischaemic aspect			Ischemic/Haemorrhagic changes Infarction			Haemorrhage/ Petechial haemorrhage			Viral Intranuclear inclusion	Microglial activation/ nodules	Neuronophagia	Haemosiderin deposition	Meningeal lymphocytes
	R	L	BS	R	L	BS	R	L	BS	R	L	BS	R	L	BS	R	L	BS	R	L	BS	R	L	BS	R	L	BS					
1	−	−	−	+	+	−	+	+	−	−	−	−	+	+	+	−	−	−	+	+	+	+	−	−	−	−	−	−	−	−	+	+
2	−	−	−	−	+	−	+	+	−	−	−	−	+	+	−	−	−	−	+	+	+	−	+	−	−	−	−	−	−	−	+	+
3	+	+	+	−	−	−	−	+	−	−	−	−	+	+	−	−	−	−	+	+	+	−	+	−	M	M	F	−	−	−	+	−
4	−	−	−	−	−	−	+	+	−	−	−	−	+	+	−	−	−	−	+	+	−	−	−	−	−	F	−	−	−	−	+	−
5	+	+	−	−	−	−	+	+	+	−	−	−	−	−	−	−	−	−	+	+	+	−	−	−	F	F	−	−	−	−	+	−
6	−	NA	NA	−	NA	NA	+	NA	NA	−	NA	NA	−	NA	NA	−	NA	NA	+	NA	NA	−	NA	NA	−	NA	NA	−	−	−	−	−
7	−	−	−	−	−	−	+	+	−	−	−	−	−	+	−	−	−	−	+	+	−	−	−	−	−	−	−	−	−	−	+	+
8	−	−	NA	+	+	NA	+	+	NA	−	−	NA	−	−	NA	−	−	NA	+	+	NA	−	−	NA	−	−	NA	−	−	−	+	+
9	−	−	−	−	−	−	+	+	−	−	−	−	−	−	−	−	−	−	+	+	−	−	−	−	−	F	−	−	−	−	+	+
10	−	−	−	−	−	−	+	+	−	−	−	−	−	−	−	−	−	−	+	+	−	−	−	−	F	−	−	−	−	−	+	−
11	−	−	NA	+	+	NA	+	+	NA	−	−	NA	−	−	NA	−	−	NA	+	+	NA	−	−	NA	−	−	NA	−	−	−	−	−
12	−	−	NA	+	+	NA	+	+	NA	−	−	NA	−	−	NA	−	−	NA	+	+	NA	−	−	NA	−	−	NA	−	−	−	−	−
13	−	−	−	+	+	−	+	+	+	−	−	−	−	−	−	−	−	−	+	+	−	−	−	−	−	−	−	−	−	−	+	−
14	−	−	−	+	+	−	+	+	+	−	−	−	−	−	−	−	−	−	+	+	−	−	−	−	−	−	−	−	−	−	+	−
15	−	−	−	+	+	−	+	+	−	−	−	−	−	−	−	−	−	−	+	+	−	−	−	−	−	−	−	−	−	−	−	−
16	−	−	−	+	+	−	+	+	−	−	−	−	−	−	−	−	−	−	+	+	−	−	−	−	F	−	−	−	−	−	−	−
17	−	−	−	−	−	−	+	+	−	−	−	−	−	+	−	−	−	−	+	+	−	−	−	−	−	−	−	−	−	−	−	+
18	−	−	−	+	+	−	+	+	−	−	−	−	−	−	−	−	−	−	+	+	−	−	−	−	−	−	−	−	−	−	+	−

### Postmortem procedure

The Belgian Public Health Institute (Sciensano) guidelines were integrated into our postmortem procedure [18]. To ensure the safety of our autopsy team/staff, it was decided that a delay of a minimum of 48 h between death and autopsy should be sustained. The cadavers were kept in the refrigerator at 4 °C, and autopsies were performed 50 to 111 h after death. The personal protective equipment consisted of two superposed disposable latex gloves, plastic sleeves, FFP3 mask, scrub hat, clear face visor, surgical gown plus plastic apron, and rubber boots. To allow safe decontamination, the postmortem was separated into “soiled” and “clean” subsections. Using standard surgical pathology processing, complete sets of tissue samples were collected for diagnosis and biobanking. The material was biobanked by Biobanque Hôpital Erasme-ULB (BE_BERA1), CUB Hôpital Erasme; BBMRI-ERIC. For each autopsied patient, eight brain regions (right and left cerebral lobes (right brain resection, left brain resection, right stereoanterior, right stereoposterior, left stereoanterior, left stereoposterior), brainstem and dura-mater) were collected. For safety reasons, complete brain removal was not allowed, but a neurosurgeon performed a safe procedure with drills and protective devices to avoid aerosolized virus exposure in 18 cases, and 14 to 49 samples from different brain regions were obtained, as previously described by Myriam et al*.* [2]. For the lungs, we collected six samples per lobe (i.e., a total of 30 samples), except for two patients who had previously undergone lobectomy for cancer and from whom only 18 samples were taken. Formalin-fixed paraffin-embedded (FFPE) tissues underwent standard processing to provide haematoxylin and eosin (H&E)-stained sections and immunohistochemistry analysis.

### Morphological analysis – digital pathology

H&E-stained slides were digitally scanned (× 40 magnification) using a Nanozoomer 2.0 HT slide scanner (Hamamatsu, Hamamatsu City, Japan), and morphological analysis was performed by three neuropathologists (LL, IS, MBL) using a digital pathology system (SecundOs digital platform (TribVnHealth Care, Chatillon, France)). For each case, the following neuropathological features were evaluated (presence/absence): oedema, lymphocyte infiltration, perivascular haemosiderin-laden macrophages, vasculitis, arteriosclerosis of small vessels of white matter, and intravascular thrombosis. We also reported hypoxic-ischaemic aspects of neurons, ischaemic infarction, petechial haemorrhage and signs of viral infection, such as microglial activation, neuronophagia and viral intranuclear inclusion. The evaluation of meninges included the presence of haemosiderin deposition and lymphocyte infiltration. Based on the most altered samples, we selected eight brain samples and one lung sample for further IHC and qRT‒PCR analysis for each patient.

### SARS-CoV-2 detection by immunohistochemistry

Automated IHC on 4 µm-thick FFPE brain and lung sections using SARS-CoV/SARS-CoV-2 Nucleocapsid (Sino Biological, 40,143-R019, clone 019, dilution 1:10,000) and Spike (GeneTex, GTX632604, clone 1A9, dilution 1:100) antibodies were processed on Dako Omnis (Agilent Technologies, Santa Clara, CA, USA). Negative tissue controls were obtained from patients who had an autopsy before the COVID-19 pandemic. IHC evaluation was performed by three pathologists (LL, SD, IS) as follows: positive (+) and negative (−), i.e., no staining or scattered positive cells. Additional information is available in Additional file 4.

## Sequential chromogenic immunohistochemical multiplex (SCIM) to evidence SARS nucleocapsid/NeuN/CD31/CD68 coexpression

For one case (case 10), tissue sections were sequentially immunolabelled with four different antibodies: SARS-CoV-2 nucleocapsid antibody (Sino Biological, clone 019), NeuN antibody (Sigma‒Aldrich, clone A60), CD31 antibody (Dako, clone JC70A) and CD68 antibody (Dako, clone KP1). Automated IHC was processed on a PT Link and an Autostainer Link 48 (Agilent Technologies, Santa Clara, CA, USA), which was previously detailed [19, 20].

To determine whether SARSn colocalizes with CD68, CD31 or NeuN, Visiopharm DP 2021.02 software (Visiopharm, Hoersholm, Denmark) was used on the whole slide images obtained through the digitization steps outlined above. Briefly, Visiopharm’s “TissueAlign” module was used to register pairs of virtual slides targeting SARSn and each of the other markers of interest. A simple intensity threshold on the corresponding colour-deconvoluted channels was then applied to label any positively stained pixels, and colocalization was measured using intersection over union (IoU), defined as the ratio by area of positively stained overlapping pixels over the sum of positively stained (overlapping and nonoverlapping) pixels.

### SARS-CoV-2 detection by one-step qRT‒PCR

Total nucleic acid was extracted from FFPE tissues using the Maxwell RSC DNA FFPE Kit (Promega Corporation, Madison, WI, USA) and the Promega Maxwell extractor. One-step RT‒PCR assays for the SARS-CoV-2 E envelope protein gene were performed as previously described [2]. A clinical sample that was highly positive for SARS-CoV-2 (by IHC and qPCR) was diluted 1:1000 and included as a positive control. Clinical samples from before the COVID-19 pandemic were used as negative controls. For each analysis, a standard dilution curve was included for quantification purposes (2019-nCoV Charité/Berlin RUO Plasmid Controls, Integrated DNA Technologies). An internal control targeting beta actin was also included to check RNA integrity and avoid false-negatives (Assay ID Hs99999903_m1, Cat. 4,448,484, Life Technologies).

### Review of literature

A systematic literature review was conducted from the beginning of the pandemic in 2019 until June 1st, 2022. The review was performed in the PubMed database. The search terms including “SARS-CoV-2” and “COVID-19” crossed with “brain autopsy”, “brain postmortem” and “brain samples”, and only English studies were considered. Research that included at least five brain samples with microscopic examination and/or postmortem PCR and/or IHC on brain samples were research that we considered eligible for our review. PCR/IHC results of the olfactory bulb, olfactory mucosa, and olfactory tubercle were not included. Preprints were not included. In total, 586 brains were analysed by optical microscopy, 300 by PCR and 183 by IHC. For each term selected: “sars-cov-2 brain autopsy” = 116, “sars-cov-2 brain postmortem” = 139, “sars-cov-2 brain samples” = 350, “sars-cov-2 CSF samples” = 65, “covid-19 brain autopsy” = 129, “covid-19 brain postmortem” = 154, “covid-19 brain samples” = 592, and “covid-19 CSF samples” = 80. A total of 37 articles containing 699 brains were included: 29 articles contained microscopic examination and 23 articles contained PCR and/or IHC analyses (15 articles contained both analyses).

### Statistical analyses

The number of viral genome copies between the different anatomical regions was tested with the Kruskal‒Wallis Test Calculator, followed by post hoc Dunn's test. Correlation between the number of viral genome copies or the number of positive regions in the brain vs. lung regions was tested with the Kendall rank correlation coefficient. Statistical analyses were performed using RStudio v. 2022.02.0 and R v.4.2 [21].

## Results

### Study cohort

The main characteristics of the study cohort (12 males out of 18; median age, 59.5 years (46–75) are provided in Additional file 1: Table S1. The time period between the first symptoms and death ranged from 0 to 40 days (median, 20.5 days). All patients had at least one comorbidity, the most frequent being obesity (n = 13), hypertension (n = 13), dyslipidaemia (n = 8), diabetes (n = 7), coronary artery disease (n = 7), chronic pulmonary disease (n = 6), cancer (n = 5), cerebrovascular disease (n = 4), chronic neurological disorder (n = 4), and chronic renal disease (n = 4). None of the patients had tested positive on admission for respiratory syncytial virus or influenza A and B viruses. Seven out of 18 patients were treated with ECMO, and 15 of 18 also benefited from MV. Other treatments included, starting from the most frequent, antibiotic therapy (n = 17), unfractionated heparin (n = 14), enoxaparin (n = 13), hydroxychloroquine (n = 12), corticosteroid (n = 9), lopinar-ritonavir (n = 8), acetylsalicylic acid (n = 6) and oseltamivir (n = 4). The reported causes of death were respiratory failure (n = 6) followed by multiple-organ failure (MOF) (n = 10), sudden cardiac arrest (n = 1) and septic shock concomitant to mesenteric ischaemia (n = 1).

### Neuropathological features

**Fig. 1:**
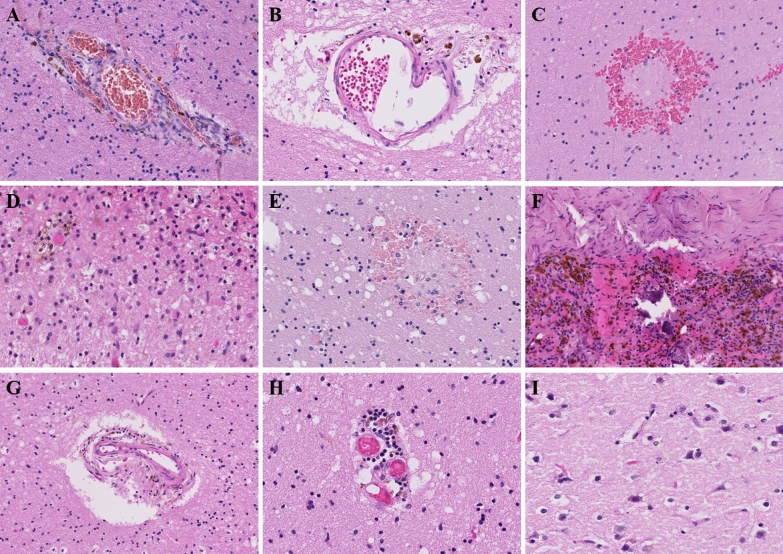
Neuropathological findings in the postmortem brain samples of the COVID-19 patients in our series (n = 18). **A**, **B** Perivascular haemosiderin-laden macrophages (Case 9-left hemisphere)(HE, magnification 200X) **C** White matter petechial haemorrhage (Case 16-right hemisphere)(HE, magnification 200X) **D** Subacute infarction-like white matter with macrophagic reaction, gliosis and haemosiderin deposition (Case 2-left stereoposterior)(HE, magnification 200X) **E** White matter oedema with petechial haemorrhage (Case 5-right stereoanterior)(HE, magnification 200X). **F** Meningeal lymphocytes and haemosiderin deposition (Case 2-dura-mera)(HE, magnification, 100X). **G** Arteriosclerosis of small vessels of white matter (Case 1-right hemisphere)(HE, magnification, 100X). **H** Perivascular lymphocyte (Case 1-right hemisphere)(HE, magnification, 200X). **I** Hypoxic-ischaemic aspect of neurons (HE, magnification, 200X). HE: Haematoxylin–eosin

As reported in Table 1 and illustrated in Fig. 1, the main histological findings included perivascular haemosiderin-laden macrophages and hypoxic-ischaemic changes in the neurons, which were found in all cases (n = 18). Other histological findings included oedema (n = 2), lymphocyte infiltration/perivascular lymphocytes (n = 10), petechial haemorrhage (n = 6) and arteriosclerosis of the small vessels of the white matter (n = 6). Three patients harboured focal ischaemic infarction without identified intravascular thrombosis. Meningeal haemosiderin deposition was also noted in 12 patients, with some patients harbouring meningeal lymphocytes (n = 6). None of the patients harboured vasculitis or viral infectious changes (viral inclusions, microglial nodules, microglial activation and neuronophagia).

## SARS-CoV-2 detection in brain samples and comparison to SARS-CoV-2 detection in lung samples using PCR and IHC

### SARS-CoV-2 detection using PCR

**Fig. 2:**
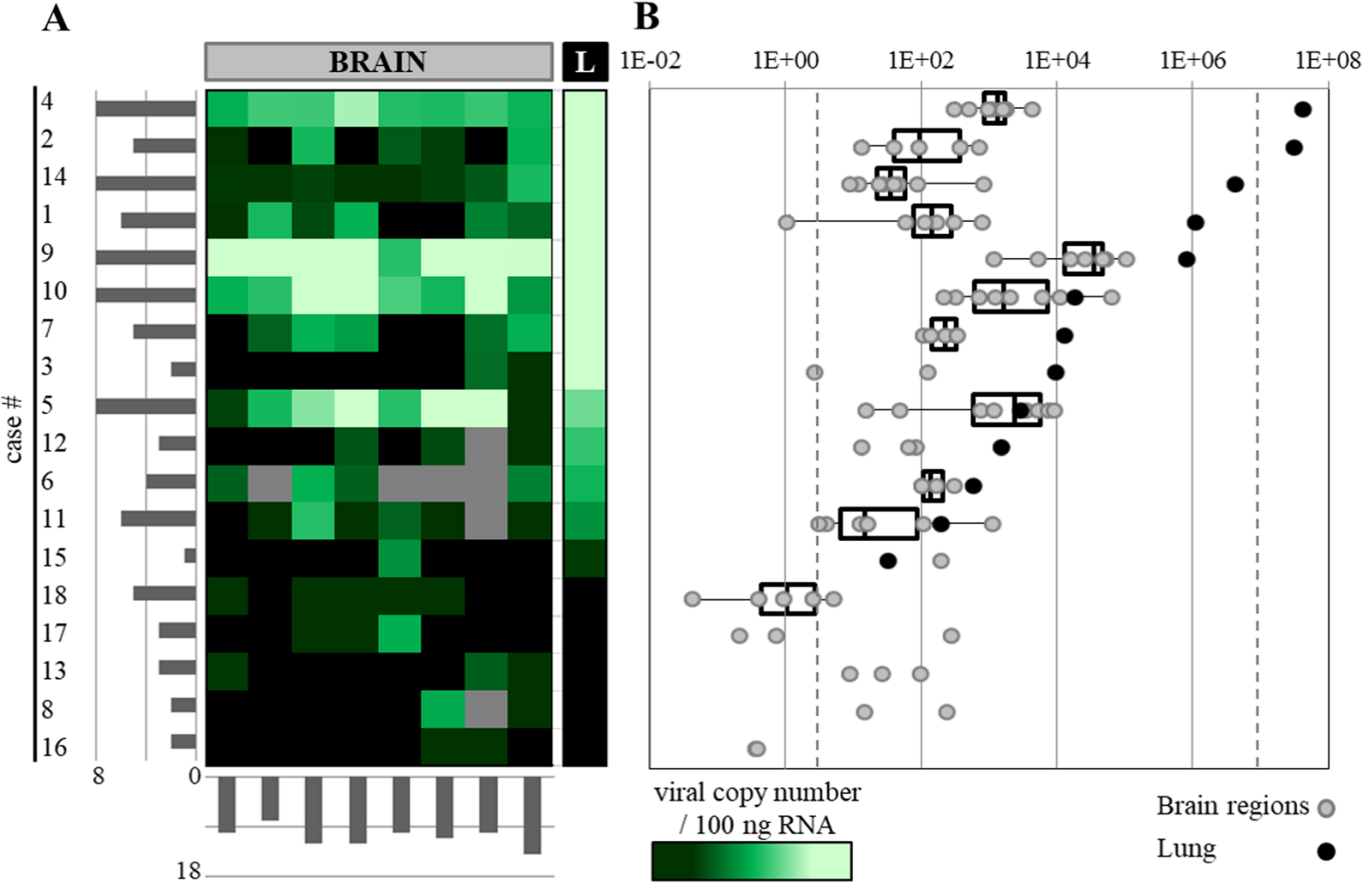
Graphical representation of the SARS-CoV-2 viral genome and qRT‒PCR values obtained from the different brain and lung regions. **A** VCN is represented on a coloured scale (black = not detected, light green = high expression, grey = material not available). The patients are shown in each row and sorted by the VCN value in their lung sample (« L» column, top to bottom as higher to lower values). The lateral bars indicate the number of brain regions for each patient where the viral genome was detected (y-axis) or the total number of patients showing the presence of the viral genome for each specific region (x-axis). Order of the tested brain regions from left to right: right brain resection, left brain resection, right stereoanterior, right stereoposterior, left stereoanterior, left stereoposterior, brainstem, dura-mater. **B** VCN values in a box-plot representation showing the limits of the 1st quartile, median and 3rd quartile, whiskers showing min/max values (black dots = lung, grey dots = brain regions, same vertical order as in **A**, x-axis showing exponential VCN increase with dashed lines marks validated standard curve limits)

The PCR results regarding the detection of SARS-CoV-2 RNA are summarized in Fig. 2.

In this study, different areas of the brain were sampled at the moment of autopsy and were later tested by qRT‒PCR. When combining all the results, the presence of SARS-CoV-2 RNA in the brain was confirmed in all autopsies performed in this study (n = 18). Nevertheless, as can be observed in Fig. 2, the brain regions present a heterogeneous pattern of positivity with some patients having detection in all (or almost all) regions and some others in just a minority of the samples. For example, patient 15 had just one region with a positive PCR. In the lung samples, SARS-CoV-2 RNA was not detected in five samples (cases 8, 13, 16, 17, 18). In this study, we included data from samples falling out of our standard curve limits (Fig. 2, dashed vertical lines), mainly because consistent amplification curves with a proper sigmoid shape were observed at late cycles (this can be explained, for example, by inhibitory molecules in the starting material). If we consider all the PCR results from this study (by grouping them all together), we have a global positivity rate of 56–64% (76 to 87 positive samples of a total of 137 tested regions, depending on whether we consider values outside the standard curve (11 samples)). Different brain regions showed an individual positivity rate between 47% (8/17 for left brain resection) and 78% (14/18 for the dura mater). Viral copy number (VCN) is not significantly different between the different tested regions (the Kruskal‒Wallis H test indicated that there is a nonsignificant difference in the dependent variable between the different groups, χ2(8) = 4.3, p = 0.829). We observed a trend when comparing the VCN present in the lungs and the positivity in distinct brain regions: patients with higher VCN values from lung tissue usually have more positive CNS regions (the results of Kendall's rank correlation indicated that there is a significant medium positive relationship between lung VCN and the number of positive brain regions, (r = 0.449, *p* = 0.025)). In addition, the VCN in the brain seemed to also correlate with the VCN in the lungs (the results of Kendall's rank correlation indicated that there was a significant large positive relationship between the lung VCN and mean brain VCN, (r = 0.504, *p* = 0.006)).

### SARS-CoV-2 detection using immunohistochemistry

**Fig. 3:**
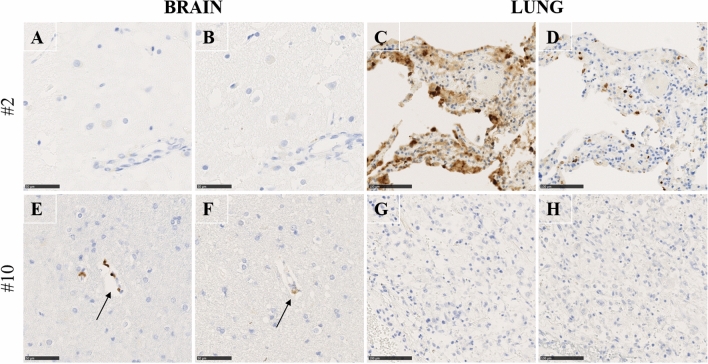
Immunohistochemical detection of SARS-CoV-2. Immunohistochemical detection of SARS-CoV-2 using antibodies targeting nucleocapsid (**A**, **E**, **C**, **G**) and spike proteins (**B**, **F**, **D**, **H**). Left panels showing brain regions (**A**-**B** left stereoanterior, **E**–**F** right stereoanterior); right panels showing lung regions (**C**, **D**, **G**, **H**). One representative COVID-19 case per line: Case 2 (A-D) and Case 10 (E–H). Case 2: No viral proteins were detected in the brain parenchyma **A**, **B**) while viral proteins were detected in the lung (**C**, **D**). Case 10: viral proteins were detected near some brain capillaries (arrows) (**E**, **F**) while viral proteins were not detected in the lung (**G**, **H**). Brain, Scale bar = 50 µm; Lung, Scale bar = 100 µm

**Fig. 4:**
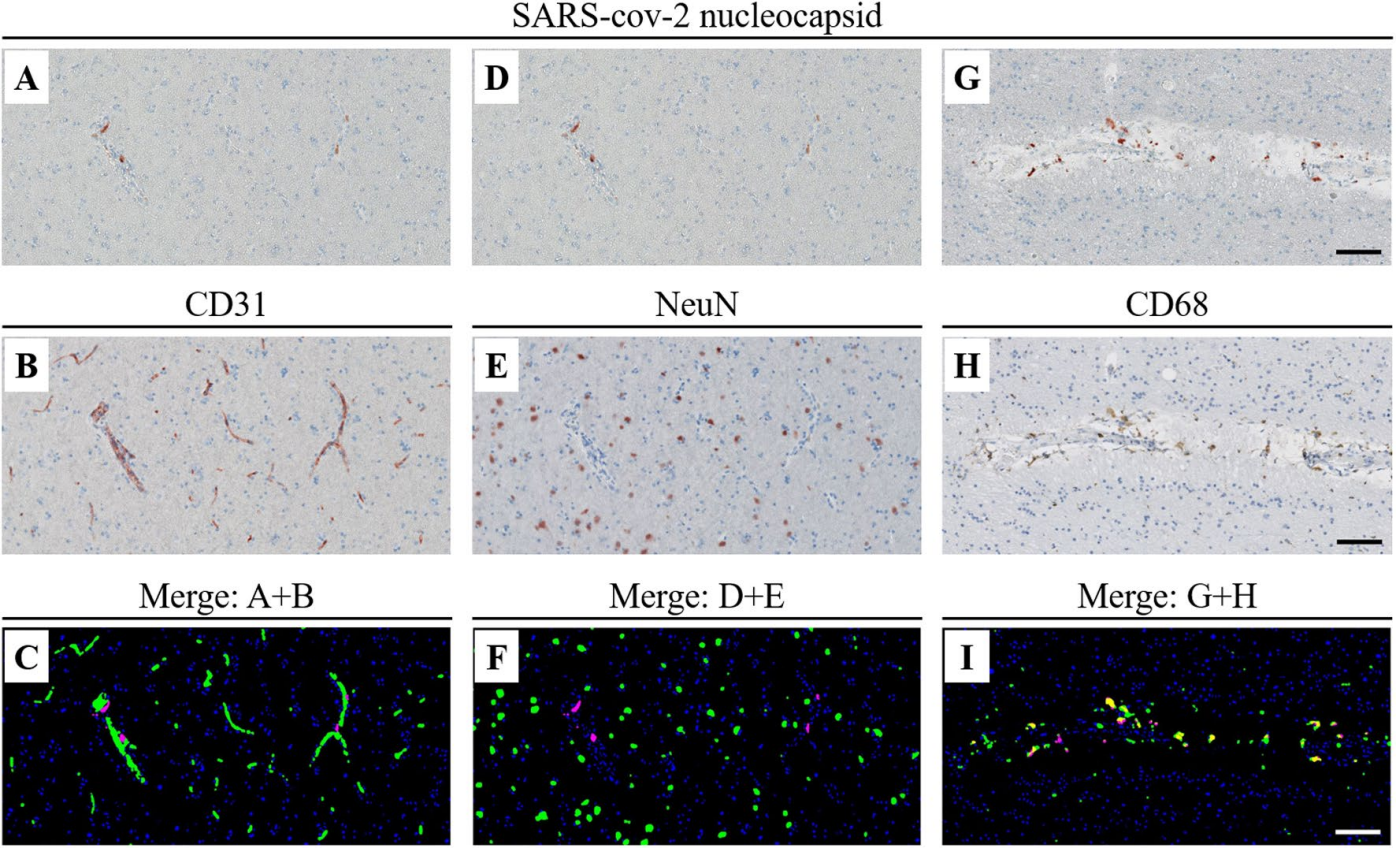
Immunohistochemical colocalization of SARS-CoV-2 with CD31, NeuN and CD68. Immunohistochemical colocalization of SARS-CoV-2 using SARS-CoV-2 nucleocapsid (**A**, **D**, **G**) by sequential immunochemistry of CD31 (**B**), NeuN (**E**) and CD68 (**H**) in the brain samples of Case 10 (right hemisphere). **C**: digital overlap of **A** (nucleocapsid in magenta) and **B** (CD31 in green). **F** digital overlap of **D** (nucleocapsid in magenta) and **E** (NeuN in green). **I** digital overlap of **G** (nucleocapsid in magenta) and **H** (CD68 in green). Coexpression is noticeable only in **I** (nucleocapsid and CD68, in yellow pseudo-color). Scale bar = 100 µm

The immunohistochemistry results are summarized in Figs. 3 and 4 and Additional file 2: Table S2. An immunohistochemical analysis was performed to detect SARS-CoV-2 viral spike or SARS-CoV-2 nucleocapsid proteins in the brain and lung from 18 patients with confirmed SARS-CoV-2 infection. One brain and one lung tissue block per patient were selected based on the morphological analysis results. Two more brain samples per patient, selected based on the qRT‒PCR results (lowest Cp values), were analysed if available. While both SARS-CoV-2 spike and nucleocapsid proteins were detected in the cells of the lung parenchyma in 5 out of 18 patients, no similar staining pattern was observed in the brain samples of all patients (Fig. 3 and Additional file 2: Table S2). Of note, for one particular patient (case 10), SARS-CoV-2 viral spike and nucleocapsid proteins were present at low levels near some brain capillaries (Figs. 3E, F, arrow and Fig. 4A, D, G) but were absent in the lung (Fig. 3G, H). As controls, no staining was detected with the secondary antibody only, and Perls staining appeared negative for haemosiderin deposits (Additional file 5: Fig. S1). To investigate in which cells SARS-CoV-2 antigens were present, we performed staining for endothelial cell (CD31), neuron (NeuN) and macrophage (CD68) detection (Fig. 4 B, E, H). Quantification analysis allowed us to highlight that there was coexpression of CD68 and SARSn, while there was no coexpression of NeuN and CD31 (Fig. 4C, D, I). Therefore, this led to the hypothesis that SARS-CoV-2 nucleocapsid proteins were present in macrophages in the Virchow-Robin spaces.

## Literature review and comparison with our study (PCR and/or IHC in brain for at least 5 cases)

A total of 37 studies including microscopic examination and/or PCR and/or IHC on brain samples associated with SARS-CoV-2 infection were included and summarized in Table 2. Twenty-three studies used at least one of these techniques (PCR, IHC), 19 studies used PCR, 10 studies used IHC, and 6 studies used both techniques.

**Table 2:** Neuropathological findings in the postmortem brain samples of the COVID-19 patients in the literature

Reference number	Brains	Microscopic examination (≥ 5)	Inflammatory changes			Thrombotic/Arteriosclerosis changes		Ischemic/Haemorrhagic changes			Viral changes			Cerebral presence of SARS-CoV-2 (≥ 5)	
			Oedema	Perivascular lymphocyte	Vasculitis	Arteriolosclerosis/Atherosclerosis	Thrombosis/Microthrombosis	Hypoxic-ischaemic aspect	Ischaemic/ Haemorrhagic Infarction	Haemorrhage/ Petechial haemorrhage	Viral Intranuclear inclusion	Microglial activation/nodules	Neuronophagia	PCR	IHC
[22]	8	Y	−	+	−	−	+	+	+	−	−	+	−	NA	
[23]	5	Y	−	−	−	−	−	−	−	+	−	−	−	NA	
[24]	58	Y	+	+	−	−	+	−	+	+	−	−	−	NA	
[25]	10	Y	−	+	−	−	−	−	+	+	−	−	+	NA	
[6]	7	Y	+	−	−	+	−	+	−	−	−	+	−	Y	NA
[26]	11	NA	NA			NA		NA			NA			Y	NA
[27]	9	Y	−	−	−	−	−	−	−	+	−	−	−	NA	
[28]	9	Y	−	−	−	−	+	+	+	−	−	−	−	NA	Y
[29]	33	Y	+	−	−	−	+	+	+	+	−	+	−	Y	NA
[10]	9	Y	+	+	−	+	−	−	−	−	−	+	−	Y	NA
[30]	15	NA	NA			NA		NA			NA			NA	Y
[31]	7	Y	−	−	−	+	−	−	+	−	−	−	−	NA	
[32]	5	Y	+	+	−	−	−	+ *	−	−	−	+	−	Y	NA
[33]	15	Y	+	−	−	+	−	+ *	−	+	−	−	−	Y	NA
[34]	20	Y	−	−	−	−	−	−	−	−	−	−	−	NA	
[35]	45	Y	+	−	−	−	+	−	−	−	−	−	−	NA	
[36]	21	Y	−	−	−	−	−	−	−	+	−	+	−	NA	
[8]	18	Y	+	+	−	−	−	+ *	+	+	−	+	−	NA	
[37]	12	Y	+	−	−	−	+	−	−	+	−	−	−	NA	
[38]	6	Y	+	−	−	−	+	+	−	+	−	+	−	Y	Y
[16]	43	Y	+	+	−	+	−	+	+	−	−	+	+	Y	Y
[11]	27	Y	−	−	−	−	−	+	+	+	−	+	−	Y	Y
[39]	13	Y	+	−	−	−	+	−	−	−	−	−	−	NA	Y
[40]	33	NA	NA			NA		NA			NA			Y	NA
[41]	7	NA	NA			NA		NA			NA			Y	NA
[42]	21	NA	NA			NA		NA			NA			Y	NA
[2]	11	Y	+	−	−	−	−	−	+	+	−	−	−	Y	NA
[43]	44	Y	−	+	+	−	+	−	+	+	−	+	−	NA	
[44]	9	Y	−	+	−	−	−	+	−	−	−	+	−	NA	Y
[45]	25	Y	−	+	−	−	−	−	−	−	−	+	−	Y	Y
[46]	7	NA	NA			NA		NA			NA			Y	NA
[47]	18	Y	−	+	−	+	−	+	−	−	−	+	−	Y	Y
[12]	41	Y	−	+	−	+	+	+	+	+	−	+	+	Y	Y
[48]	6	Y	−	+	−	−	−	−	−	+	−	−	−	NA	
[49]	12	NA	NA			NA		NA			NA			Y	NA
[50]	52	Y	−	+	−	−	−	−	−	+	−	+	−	NA	
[51]	7	NA	NA			NA		NA			NA			Y	NA
TOTAL	699	586 (29 studies)	13	14	1	7	10	13	11	16	0	16	3	19	10

### Microscopic examination of the brain samples associated with SARS-CoV-2 infection

Table 2 summarizes all the neuropathological changes described in the literature. To date, 29 studies have reported microscopic examination of brain samples associated with SARS-CoV-2 infection. Grouping together, 586 brain samples were microscopically examined, ranging from 5 to 58 samples per study, while 384 brain samples were analysed by PCR and/or IHC. Regarding the most frequent lesions described, ischaemic and haemorrhagic lesions were the most frequently reported (80%, 24/30), and the lesions included hypoxic/ischaemic alterations, haemorrhage/microhaemorrhage and ischaemic/haemorrhagic infarction. Inflammatory changes were also frequently described (73%, 22/30), such as oedema and perivascular inflammation, including haemosiderin-laden macrophages. Another frequently observed morphological alteration is microglial activation (53%, 16/30), and 10 studies out of 30 (33%) reported thrombosis/microthrombosis. Perivascular lymphocytes and intranuclear inclusions were rarely described.

### SARS-CoV-2 detection by PCR

A brief analysis of Table 3 reveals that in the various studies, the samples used for PCR analysis were evenly represented by cryopreserved, freshly used and/or FFPE material (6, 6 and 7 studies, respectively). The majority of the studies used the same target in the SARS-CoV-2 genome, i.e., the envelope (E) gene (8 studies); the second most frequently used target was the ORF1ab gene (5 studies, in 2 of which it was coupled with spike (S) and nucleocapsid (N) genes) and less frequently analysed the nucleocapsid (N) genes (4 studies). In Matschke et al. [16] as well as in Puelles et al. [42], a molecular setup similar to our study, using the same target genes and internal control system as Matschke and colleagues obtained a 50% positivity rate from FFPE material (4 positive samples out of 8 tested), which is comparable to what we obtained [16]. A detailed list of PCR targets used in these articles is listed in Additional file 3: Table S3.

**Table 3:**
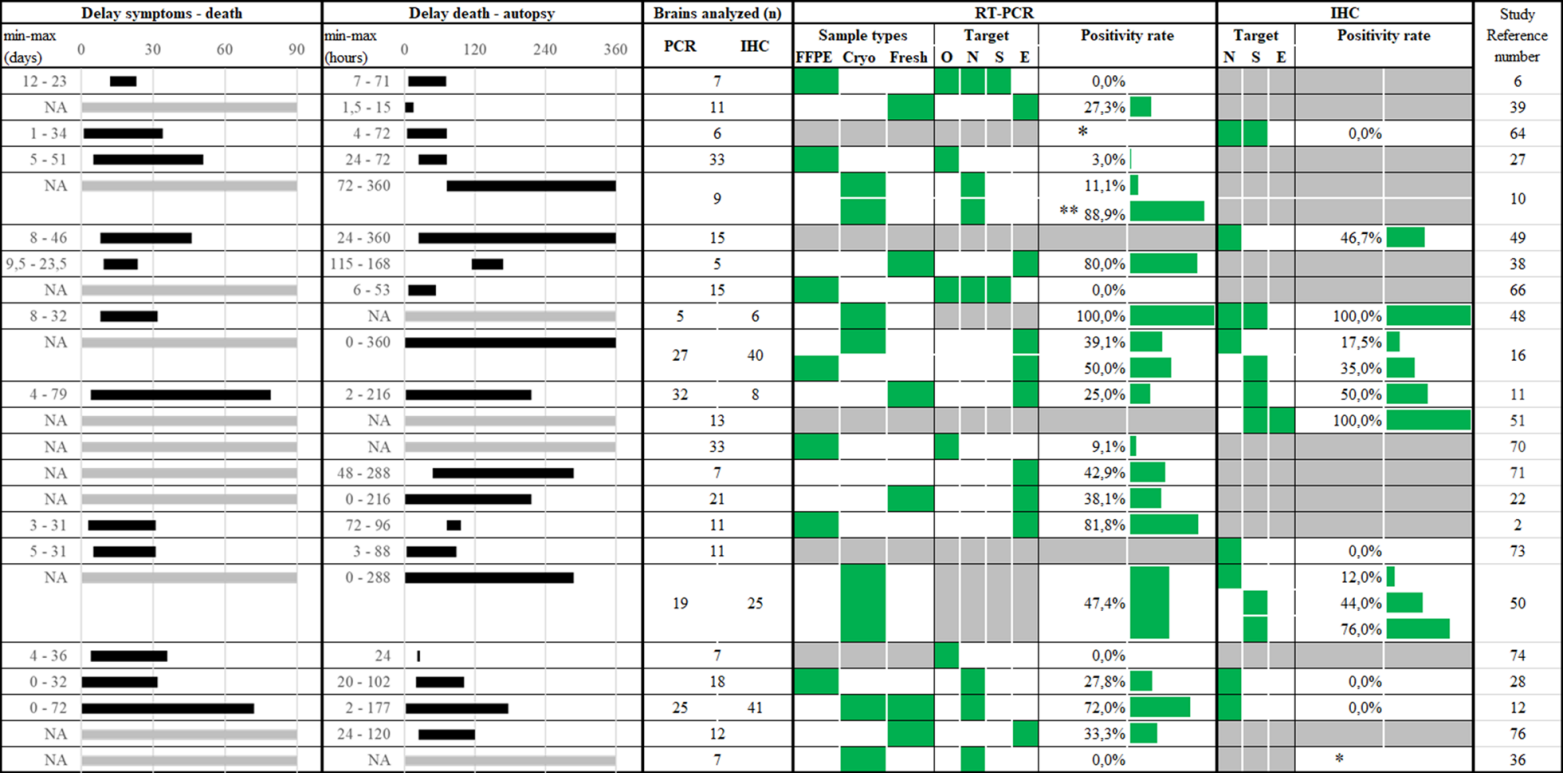
SARS-CoV-2 PCR and immunohistochemistry in the postmortem brain samples of the COVID-19 patients in the literature

Green bars visual representation of positivity rate percentages values. Grey filled cells and bars/NA: data not available. Cryo: Cryopreserved/frozen tissue. Fresh: fresh tissue. *less than 5 brains analyzed so not taken into consideration for this figure. **RT-ddPCR. O ORF1ab. N N gene/protein. S S gene/protein. E E gene/protein

### SARS-CoV-2 immunohistochemistry

To date, approximately 10 articles (Table 3) have reported SARS-CoV-2 immunohistochemistry (IHC) performed on brain samples from COVID19-infected patients (with at least five cerebral cortex/brainstem samples tested).

The number of cases per article on which anti-SARS-CoV-2 IHC was performed varied from 6 to 41 (< 10 (n = 3 studies) and > 10 (n = 7 studies)). Some research focuses on one area of the brain (n = 2). and others focused on several samples (n = 8). Samples from the cerebral cortex, brainstem or both were analysed in three, two and six studies, respectively.

Manual (n = 7) or automated (n = 4) IHC was performed using one (n = 4) or several (n = 6) antibodies against SARS-CoV-2 proteins. The different antibody targets consisted of the nucleocapsid (n = 7), spike (n = 4), envelope (n = 1) and membrane (n = 1) proteins of SARS-CoV-2. At least seven different antibodies have been referenced in these articles for nucleocapsid protein and four for spike protein. The most frequently employed antibody for the nucleocapsid protein was Sino Biological, 40,143-R001, clone 001 (n = 3).

Regarding the detection of viral proteins in brain samples from COVID-19-positive patients, negative results were concluded in four studies. In the others, positivity was mainly described in endothelial cells (n = 5) within the cerebral cortex and brainstem. SARS-CoV-2 proteins are also described in isolated cells near capillaries (n = 3) and microglial cells (n = 2). Positivity for SARS-CoV-2 viral proteins was detected by anti-spike (n = 7), anti-nucleocapsid (n = 6), anti-envelope (n = 1) and anti-membrane (n = 1) antibodies using manual (n = 5) or automated (n = 2) IHC. The positivity rate of the samples per study ranged from 12 to 100%.

A detailed list of the antibodies used in these articles is listed in Additional file 3: Table S3.

## Discussion

SARS-CoV-1 and MERS-CoV have already been associated with neurotropism and ensuing neurological disorders such as stroke, seizures and encephalitis for SARS-CoV-1 and confusion, seizures, acute disseminated encephalomyelitis (ADEM), encephalitis, infarcts, Guillain‒Barré syndrome (GBS) and neuromuscular disease for MERS-CoV. In contrast, neuropathological findings in patients with SARS-CoV-2 infection remain limited, with neurological injuries such as vasculitis, intravascular coagulation, and thromboembolic events suggested to be linked to the subsequent inflammatory responses of the virus infection [14]. This difficulty relies on confounding factors such as drugs and supportive treatments as well as the comorbidities that these infected patients are typically associated with. Herein, we report our experience, integrated in an extensive review of the literature regarding the neuropathological findings and detection of SARS-CoV-2 in postmortem brain samples.

### Brain pathological lesions and SARS-CoV-2 infection

As shown in our literature review, most studies reported pathological lesions associated with SARS-CoV-2 infection, with ischaemic and haemorrhagic injury being the most frequent. However, none specifically reported a causal effect between viral infection and these pathological alterations. Indeed, most of the described lesions are nonspecific findings that could be associated with the provided supportive treatments or hypoxia associated with respiratory failure. It is well known that supportive treatments such as ECMO or MV may induce neurological complications such as clinical seizures, ischaemic strokes, and intracerebral haemorrhage [52]. Cerebral haemorrhages are among the most common histological lesions reported in patients who died after ECMO in the literature. A study reported microhaemorrhages and macrohaemorrhages in 37% and 35% of patients, respectively, as well as infarctions (47%) and hypoxic-ischaemic brain injury (40%) [53]. In our study, we also noted microhaemorrhage in one-third of the patients (n = 6). All of them received ECMO and/or MV except for one patient. Moreover, the vast majority of them received enoxiparin or unfractionated heparin (n = 5). Other frequent pathological changes described included thrombosis/microthrombosis, which were found in ten studies. Some authors suggest that thrombotic complications are common in SARS-CoV-2-infected patients and may contribute to the development of neurological symptoms [54, 55].

Fabbri et al*.* suggested that ischaemic lesions could be associated with the prothrombotic potential of SARS-CoV-2 [29]. However, we considered that a direct link between these events is difficult to assess because of the high rate of comorbidity that most of the patients are associated with. In our study, none of the patients harboured thrombosis or microthrombosis, which are findings that are in line with other previous studies [6, 47], such as Solomon et al., who concluded that no specific changes associated with SARS-CoV-2 were reported [47]. According to Fabbri et al., the most common pathological lesions that we noted were perivascular haemosiderin-laden macrophages [29]. However, this feature is clearly nonspecific and can be observed in many other conditions, such as cerebral microbleeds associated with angiopathy [56] and most commonly with cerebrovascular arteriosclerosis [57]. Few reports have described primary CNS vasculitis in cases of COVID-19 infection [58], diagnosed only based on imaging with rare cases of biopsy-confirmed CNS vasculitis [59]. The underlying proposed mechanism is endotheliitis, which has been shown in other organs, such as renal and gastrointestinal organs, and is caused by the link with the angiotensin-converting enzyme 2 receptor [59]. Other severe findings in SARS-CoV-2-infected patients have been published, such as demyelination changes. Several case reports of demyelination of both peripheral and central nervous systems have been published [60] but without clear causality inference established. Some papers have discussed brain demyelination linked to COVID-19 as a potential mechanism of neurological complications [61]. At this time, there are no reports of specific viral cytological modifications, neither viral inclusions nor specific cellular changes, that are recognizable as direct viral infection [6, 29, 47, 62]. Notably, regarding microglial nodules, CD68 immunostaining was not performed on all sections, which can lead to some limitations in the detection of microglial activation based only on morphology. Finally, transcriptome analysis from 30 frontal cortex and choroid plexus samples across 14 control individuals and 8 patients with COVID-19 did not highlight molecular traces of SARS-CoV-2 in the brain [51]. Interestingly, this study observed cellular dysregulation, including barrier cells of the choroid plexus and synaptic signalling of upper-layer excitatory neurons but also COVID-19-specific microglia and astrocyte subpopulations that share pathological features that have been previously reported in human neurodegenerative disease [51]. However, it has to be elucidated how these molecular processes could contribute to COVID-19 neurological symptoms [51].

Therefore, not all described lesions could be directly linked to SARS-CoV-2 infection but to iatrogenic conditions and comorbidities. One limitation of our study is that no control population was used. However, in the literature, only one study used a control population [6] and led to the conclusion that there are no specific neuropathological abnormalities linked to SARS-CoV-2 infection because these abnormalities have alterations that have also been observed in SARS-CoV-2-negative patients.

Due to the absence of specific neuropathological changes, we therefore used complementary techniques such as IHC, PCR and electronic microscopy (data not shown) to guide us in the identification of the presence of the SARS-CoV-2 virus in brain tissue. Most of the previously reported postmortem brain studies did not compare their results in the brain with those obtained in the lung tissue. Here, we provided IHC and PCR analyses in both brain and lung tissues and compared the results acquired in these two organs.

### RT‒qPCR and SARS-CoV-2 detection

Our study is concordant with other publications on SARS-CoV-2 in the brain, notably the discrepancy between PCR and IHC techniques. As shown in Table 3, basically all studies reported positive PCR detection in the brain. Even with a wide range of positivity rates, these differences can be explained by the variety of target genes and techniques used. The sensitivity testing of our assay (Ct values and relative copy numbers) is in line with previous studies [26, 32, 63] and the original article describing the primers/probe design we used [64]. From a technical point of view, quantitative comparison of SARS-CoV-2 via PCR results is challenging. Many authors tend to compare Cq/Ct values, but this should be avoided (following the joint announcement from the Infectious Diseases Society of America and the Association for Molecular Pathology, March 2021) [65, 66]. The common problems that are found when comparing PCR data are lack of standardization in normalization, controls (regarding the quality of the engaged material and PCR) and proper quantification. There are also questions regarding the clinical relevance of RT‒qPCR results in tissues: a very low copy number detected by PCR could be a reflection of the viral genetic material that is not able to infect cells (free circulating, nonfunctional viruses, residual material inside macrophages) [37]. Interestingly, two patients (Cases 9 and 10) harboured high viral levels and were both on immune suppressive medications based on their clinical history of renal and liver transplant. It has been reported that immunodeficiency, such as T-cell deficiencies, HIV infection, immunosuppressants or chemotherapy, is associated with severe COVID-19 and a higher risk of ICU admission [67]. Regarding high viral levels, patients with immune dysregulation require more time to eliminate the virus [67, 68], which could be a hypothesis of the relationship between high viral levels and immune suppression.

In the current study, we confirmed previous results [2] and expanded the sample size of the cohort. Indeed, the previous publication was focused on regions with evident histopathological lesions, therefore leading to selection bias. In the present study, discrepancies were observed within the PCR approach between tissue samples: for five patients, SARS-CoV-2 RNA was detected in the brain but not in the lungs. This observation might be explained in different ways. First, it could be related to faster/quicker viral clearance in the lungs compared to the brain. Second, the delay between death and autopsy (between 2 and 4.5 days) could play a role in RNA detection. Finally, we could not exclude the possibility that viral RNA might have reached the brain due to the degradation of the blood‒brain barrier triggered by the death of the patient. We were able to highlight a positive correlation between the VCN in the lung tissue and the VCN/number of positive regions in the brain of individual patients.

The limitations of our design are for sure the lack of a standardization of the input material other than engaging the same amount of RNA in each reaction. A possible alternative could be to homogenize tissue and use a fixed volume for nucleic acid extraction (but that would limit the use FFPE samples) [26]. Another limitation is the integrity of the RNA in our material considering the variability and sometimes extended delay between patient death and autopsy.

### IHC and SARS-CoV-2 detection

In our study, we did not detect any viral proteins in neurons and glial cells of the brain tissue (18 patients with confirmed SARS-CoV-2 infection) despite using two different anti-SARS-CoV-2 antibodies, even in the patients with high levels of spike and nucleocapsid proteins in the lung parenchyma (5/18). These results are in line with the majority of publications regarding the distribution of SARS-CoV-2 in the CNS. To our knowledge, only a few studies have reported SARS-CoV-2 viral expression in neurons of the cerebral cortex [69], brainstem [70] and glial cells [38]. The great diversity of autopsy approaches, sample preparation, anti-SARS-CoV-2 antibodies and immunohistochemistry protocols as well as the clinical history of each patient may be one of the many causes for these divergent results.

The only sign of viral protein detection in our brain cohort was observed at low levels in one patient (1/18) but in the periphery of a few capillaries in the cortex and brainstem. Seven tissue-based studies assessing CNS alterations in fatal COVID-19 described similar SARS-CoV-2 staining in cortical and/or brainstem capillaries, mostly as an infrequent event [11, 30, 38, 39, 45, 69, 70]. SARS-CoV-2 protein expression in capillaries in multiple organs has also been described [30, 71]. Whether SARS-CoV-2 proteins are localized in endothelial cells, perivascular macrophages or extracellularly near endothelial cells in correlation with the perivascular immune system/complement pathway remains poorly described. We thus further analysed brain samples from the patient of case 10 by Sequential Chromogenic Immunohistochemical Multiplex (SCIM) and suggested that SARS-CoV-2 proteins were localized in the macrophages in the Virchow-Robin spaces (SARSn+ , CD68+). Two other studies reported colocalization analysis and suggested that viral proteins are located mainly in endothelial cells but also in scattered cells close to capillaries [39, 45]. Although the ability of SARS-CoV-2 to directly infect macrophages and endothelial cells has already been reported [71, 72], an increasing number of recent studies have highlighted the role of the inflammatory immune response mediated by the virus [8, 71] in explaining the vascular pathologies observed in some patients with COVID-19.

The discordant qRT‒PCR and IHC results for SARS-CoV-2 detection in the brain may be explained by the different sensitivity of these assays, which is higher for qRT‒PCR. Indeed, our IHC detected SARS-CoV-2 spike and nucleocapsid proteins in the lungs that had Cp values < 25 for the E gene, while in the brains, all Cp values were > 27. This discordance has been observed by other groups [12, 47]. They proposed that SARS-CoV-2 RNA might spread to brain tissue by haematogenous pathways through CNS blood vessels and haemorrhage. This could be linked to the detection of viral proteins in the capillaries of certain patients in which viral clearance by the immune system is not complete: viral RNA with low levels or without viral proteins (qRT‒PCR + ; IHC-) and viral RNA with high levels of viral proteins (qRT‒PCR + ; IHC +).

### Electron microscopy and SARS-CoV-2 detection

While IHC and PCR techniques were not able to confirm the presence of virion particles in the brain, electron microscopy should confirm the presence of these particles in the brain. In our study, no replicating virus was found by the electron microscopy analysis of the three tested samples (data not shown). In the literature, electron microscopy studies performed in the brain samples of COVID-19-infected patients are infrequent and performed on very few cases [73–77]. Moreover, controversial results have been reported. Bulfamante et al. highlighted virus-like particles in the periaxonal matrix of the medulla oblongata and in the gyrus rectus of one patient [73]. Another study showed the presence of virions in endothelial cells [74]. In contrast, in other studies, no true virions were detected in brain samples [75], medulla oblongata [29] or frontal cortex [76]. Therefore, no robust conclusion can be made regarding the presence of replicating virus in brain samples of COVID-19-infected patients.

## Conclusion

To summarize, our study and the integrated review of the literature confirmed the controversies regarding the existence of specific neurotropism of SARS-CoV-2 virus, even if some previous studies have provided the hypothesis of brain invasion by the virus. Although PCR and IHC suggested that SARS-CoV-2 viral antigens can be found, there is still no evidence of the presence of the SARS-CoV-2 virus replicating in brain tissue. Our conclusion is that, at this time, there is a lack of evidence that neurological symptoms are linked to replicating virus in the brains of COVID-19-infected patients. Additionally, the “multifactorial context”, such as comorbidities, invasive supportive treatments and indirect impact of this multiorgan viral spread, can lead to complex neuropathological alterations that still need to be clarified.

## Supplementary Information

**Additional file 1: Table S1** Characteristics of the study population. Time to death = time from first symptoms to death. M Male; F Female, rRT-PCR Reverse transcription real-time polymerase chain reaction used as diagnostic laboratory test. HT Hypertension, CVD Cerebrovascular disease, CPD Chronic pulmonary disease, RD Rheumatologic disorder, CND Chronic neurological disorder, CAD Cardiovascular disease, CRF Chronic renal failure, TIA Transient ischaemic, LEP Leukoencephalopathy, MV Mechanical ventilation, ECMO Extracorporeal membrane oxygenation, MOF Multiple organ failure, NA Not applicable

**Additional file 2: Table S2** Detection of SARS-CoV-2 by immunohistochemistryin the FFPE postmortem brain and lung samples of 18 patients. Green = Positive, Grey = Negative

**Additional file 3: Table S3** Nucleic acids targets and epitopes/antibody detail for SARS-CoV-2 PCR and immunohistochemistry in the post-mortem brain samples of the COVID-19 patients in the literature. Grey cells: Not applicable. *less than 5 brains analyzed so not taken into consideration for this table. **RT-ddPCR. O: ORF1ab. N: N gene/protein. S S gene/protein. E: E gene/protein.

**Additional file 4:** SARS-CoV-2 Immunohistochemistry: Heat-induced epitope retrieval was performed using Dako Target Retrieval Solution pH 6 for 30 min at 97 °C, followed by primary antibody incubation for 20 min at 32 °C and detection with the Dako Envision Flex detection system according to the manufacturer’s protocol. The sections were counterstained with haematoxylin.

**Additional file 5: Fig S1** SARS-CoV-2 immunohistochemistry, negative control and Perls staining of brain sections. Immunohistochemical SARS-CoV-2 nucleocapsid protein, negative control without primary antibody and Perls staining of brain sections from Case 10: viral proteins were detected near some brain capillarieswhile no haemosiderin deposits were observed. No aspecific staining was detected with the secondary antibody only. Scale bars = 50 µm.

## Data Availability

The datasets used and/or analysed during the current study are available from the corresponding author on reasonable request.

## Abbreviations

AEC+: 3-Amino-9-ethylcarbazole
CAD: Cardiovascular disease
COVID-19: Coronavirus disease 2019
CND: Chronic neurological disorder
CPD: Chronic pulmonary disease
CRF: Chronic renal failure
CSF: Cerebrospinal fluid
CVD: Cerebrovascular disease
ECMO: Extracorporeal membrane oxygenation
FFPE: Formalin-fixed paraffin-embedded
H&E: Haematoxylin and eosin
HT: Hypertension
IHC: Immunohistochemistry
LEP: Leukoencephalopathy
MOF: Multiple organ failure
MV: Mechanical ventilation
NA: Not applicable
RD: Rheumatologic disorder
RT‒PCR: Reverse transcription real-time polymerase chain reaction
SARS-CoV-2: Severe acute respiratory syndrome coronavirus 2
SCIM: Sequential Chromogenic Immunohistochemical Multiplex
CNS: Central Nervous System
TIA: Transient Ischaemic Attack
WM: White Matter
VCN: Viral Copy Number
