# Supplementary material for: SARS-Cov-2 infection and neuropathological findings: a report of 18 cases and review of the literature
Source: Acta Neuropathol Commun. 2023 May 10;11:78. doi: 10.1186/s40478-023-01566-1 (PMC10170054; doi:10.1186/s40478-023-01566-1)
Supplement: Supplementary file 1 — Additional file 1: Table S1 Characteristics of the study population. Time to death = time from first symptoms to death. M Male; F Female, rRT-PCR Reverse transcription real-time polymerase chain reaction used as diagnostic laboratory test. HT Hypertension, CVD Cerebrovascular disease, CPD Chronic pulmonary disease, RD Rheumatologic disorder, CND Chronic neurological disorder, CAD Cardiovascular disease, CRF Chronic renal failure, TIA Transient ischaemic, LEP Leukoencephalopathy, MV Mechanical ventilation, ECMO Extracorporeal membrane oxygenation, MOF Multiple organ failure, NA Not applicable [file 40478_2023_1566_MOESM1_ESM.docx]

**Supplementary Table 1: Characteristics of the study population.**

| **ID** | **Age** | **Sex** | **Comorbidities** | **Neurological**  **history** | **Hospital admission** | **SARS-CoV-2**  **diagnosis** | **Neurological**  **symptoms** | **Time to**  **death** | **Time to**  **autopsy** | **Treatments** | **Cause of death** |
| --- | --- | --- | --- | --- | --- | --- | --- | --- | --- | --- | --- |
| 1 | 68 | M | CPD  CVD  Cancer  RD  Dyslipidaemia | TIA | Dyspnoea  Cough | rt-PCR | - | 18 | 72 h | MV  Hydroxychloroquine Antibiotics Enoxaparin | Respiratory failure |
| 2 | 64 | F | Obesity  HT  CVD  CND  Cancer  Dyslipidaemia | Haemorrhagic stroke  Seizures | Myoclonic seizures | rt-PCR | - | 7 | 111h30 | MV  Hydroxychloroquine Antibiotics Enoxaparin Unfractionated heparin | Respiratory failure |
| 3 | 56 | M | Obesity  HT  CPD  CND  Cancers | LEP | Dyspnoea | rt-PCR | Headache | 13 | 68 h | MV  ECMO  Hydroxychloroquine Lopinavir-Ritonavir Antibiotics Enoxaparin Unfractionated heparin | Mesenteric ischaemia  MOF |
| 4 | 66 | M | HT  Diabetes  CAD  CPD CND  CRF  Pancreatitis Alcohol Dyslipidaemia | LEP | Thoracic pain | Antigen test | Confusional status | 13 | 80 h | Antibiotics Acetylsalicylic acid | Septic shock  MOF |
| 5 | 49 | F | Obesity  HT  Diabetes  CRF | NA | Dyspnoea | rt-PCR | Headache | 16 | 63 h | MV  ECMO  Hydroxychloroquine Lopinavir-Ritonavir Oseltamivir Antibiotics Enoxaparin Unfractionated heparin | Respiratory failure |
| 6 | 63 | M | Obesity  HT  Diabetes | NA | Dyspnoea, Alteration of the general state | rt-PCR | Hallucinations Confusional status | 27 | 72 h | MV  ECMO  Hydroxychloroquine Lopinavir-Ritonavir Oseltamivir Antibiotics Corticosteroids Enoxaparin Unfractionated heparin | Respiratory failure |
| 7 | 75 | M | Obesity  HT  Diabetes  CAD  Dyslipidaemia | NA | Dyspnoea | rt-PCR | - | 13 | 88 h | MV  Hydroxychloroquine Lopinavir-Ritonavir Oseltamivir Antibiotics Unfractionated heparin Acetylsalicylic acid | MOF |
| 8 | 61 | M | Obesity | NA | Severe dyspnoea | rt-PCR | - | 40 | 50 h | MV  Hydroxychloroquine Remdesivir Oseltamivir Antibiotics Corticosteroids Enoxaparin Unfractionated heparin | MOF  Septic shock |
| 9 | 70 | F | Obesity  HT  Diabetes  CAD  Cancer  Liver transplant | NA | Dyspnoea  Cough | rt-PCR | **-** | 27 | 65 h | MV  Valganciclovir Antibiotics Corticosteroids Enoxaparin Unfractionated heparin Acetylsalicylic acid Immunosuppressive drug | MOF  Septic shock |
| 10 | 53 | M | HT  CPD CAD  CRF  Renal transplant  CVD  CND  Dyslipidaemia | Stroke  LEP | Dyspnoea  Cough | rt-PCR | - | 22 | 75 h | MV  ECMO  Ganciclovir Antibiotics Corticosteroids Enoxaparin Unfractionated heparin Acetylsalicylic acid Immunosuppressive drug | MOF  Septic shock |
| 11 | 56 | M | CAD | NA | Cardiac arrest | rt-PCR | NA | 0 | 75 h | - | Sudden cardiac arrest |
| 12 | 58 | M | Obesity  Diabetes  CPD  CAD  Dyslipidaemia | NA | Dyspnoea | rt-PCR | - | 22 | 88h30 | MV  Hydroxychloroquine Lopinavir-Ritonavir Antibiotics Unfractionated heparin Acetylsalicylic acid | Septic shock  MOF  Mesenteric ischaemia |
| 13 | 72 | M | Obesity  HT  CAD | NA | Dyspnoea | rt-PCR | - | 33 | 56 h | MV  Hydroxychloroquine Lopinavir-Ritonavir Antibiotics Corticosteroids Enoxaparin Unfractionated heparin Acetylsalicylic acid | Septic shock  Mesenteric ischaemia |
| 14 | 70 | F | Obesity history HT  Diabetes  Cancer  CVD  RD  CRF  Pancreatitis  Dementia  Dyslipidaemia | Dementia  Subdural and subarachnoid haematoma | Dyspnoea  Confusion | Antigen test | Confusion Apathy | 7 | 56 h | Antibiotics Enoxaparin | Respiratory failure |
| 15 | 53 | M | HT  CPD  Dyslipidaemia | NA | Severe dyspnoea | rt-PCR | Vertigo  Dizziness | 35 | 91h45 | MV  ECMO  Hydroxychloroquine Lopinavir-Ritonavir Acyclovir Antibiotics Corticosteroids Enoxaparin Unfractionated heparin Danaparoid | Septic shock  MOF |
| 16 | 56 | M | Obesity | NA | Dyspnoea  Cough  Agnosia | rt-PCR | Anosmia | 19 | 70h30 | MV  ECMO  Hydroxychloroquine Antibiotics Corticosteroids Enoxaparin Unfractionated heparin | Distributive shock  MOF |
| 17 | 46 | F | Obesity  HT | NA | Dyspnoea, Nausea  Vomiting Ageusia | rt-PCR | Ageusia Dizziness | 33 | 69h25 | MV  ECMO  Antibiotics Corticosteroids Unfractionated heparin | MOF |
| 18 | 57 | F | Obesity  HT  HIV treated | NA | Dyspnoea  Cough | rt-PCR | - | 40 | 87h20 | MV  Hydroxychloroquine Lopinavir-Ritonavir Lamivudine Antibiotics Corticosteroids Enoxaparin Unfractionated heparin | Respiratory failure  Septic shock |
