# Supplementary material for: SARS-Cov-2 infection and neuropathological findings: a report of 18 cases and review of the literature
Source: Acta Neuropathol Commun. 2023 May 10;11:78. doi: 10.1186/s40478-023-01566-1 (PMC10170054; doi:10.1186/s40478-023-01566-1)
Supplement: Supplementary file 2 — Additional file 2: Table S2 Detection of SARS-CoV-2 by immunohistochemistryin the FFPE postmortem brain and lung samples of 18 patients. Green = Positive, Grey = Negative [file 40478_2023_1566_MOESM2_ESM.docx]

**Supplementary Table 2 : Detection of SARS-CoV-2 by immunohistochemistry (IHC) in FFPE post-mortem brain and lung samples of 18 patients.**

| **ID** | **Brain** | **Lung** |
| --- | --- | --- |
| 1 |  |  |
| 2 |  |  |
| 3 |  |  |
| 4 |  |  |
| 5 |  |  |
| 6 |  |  |
| 7 |  |  |
| 8 |  |  |
| 9 |  |  |
| 10 |  |  |
| 11 |  |  |
| 12 |  |  |
| 13 |  |  |
| 14 |  |  |
| 15 |  |  |
| 16 |  |  |
| 17 |  |  |
| 18 |  |  |
