# Supplementary figures and images for: SARS-Cov-2 infection and neuropathological findings: a report of 18 cases and review of the literature
Source: Acta Neuropathol Commun. 2023 May 10;11:78. doi: 10.1186/s40478-023-01566-1 (PMC10170054; doi:10.1186/s40478-023-01566-1)

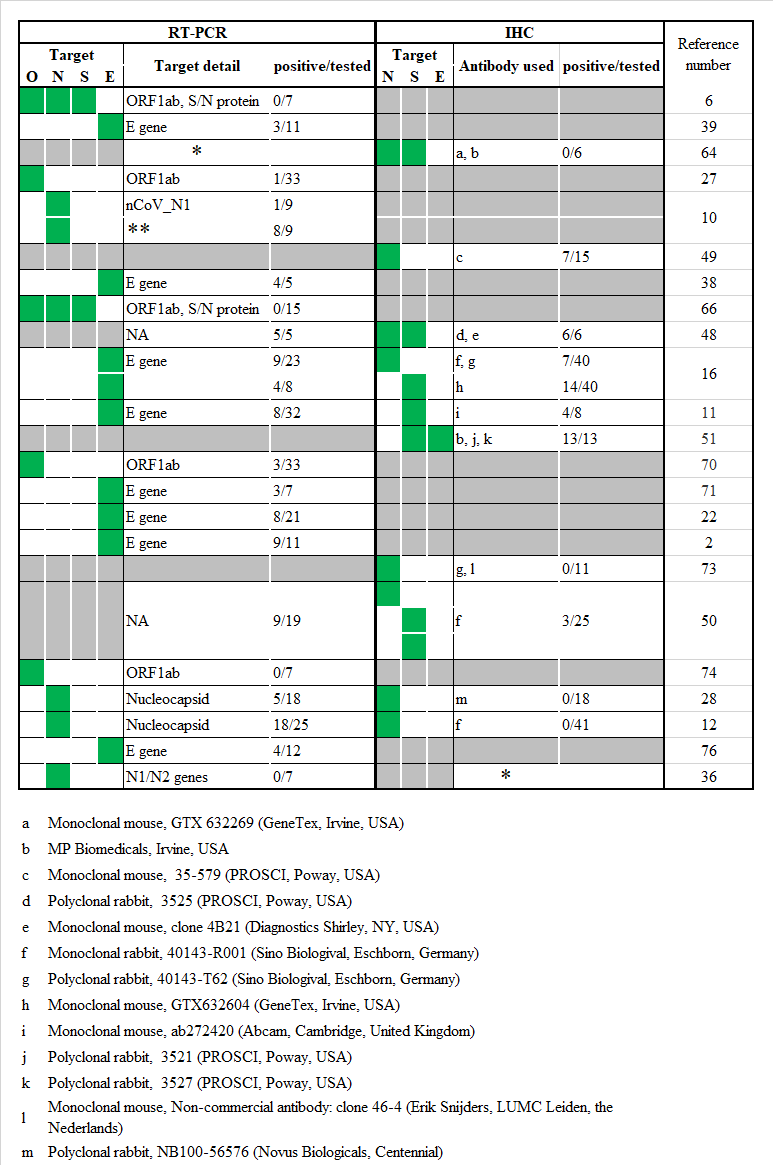

Supplement: Supplementary file 3 — Additional file 3: Table S3 Nucleic acids targets and epitopes/antibody detail for SARS-CoV-2 PCR and immunohistochemistry in the post-mortem brain samples of the COVID-19 patients in the literature. Grey cells: Not applicable. *less than 5 brains analyzed so not taken into consideration for this table. **RT-ddPCR. O: ORF1ab. N: N gene/protein. S S gene/protein. E: E gene/protein. [file 40478_2023_1566_MOESM3_ESM.tif]

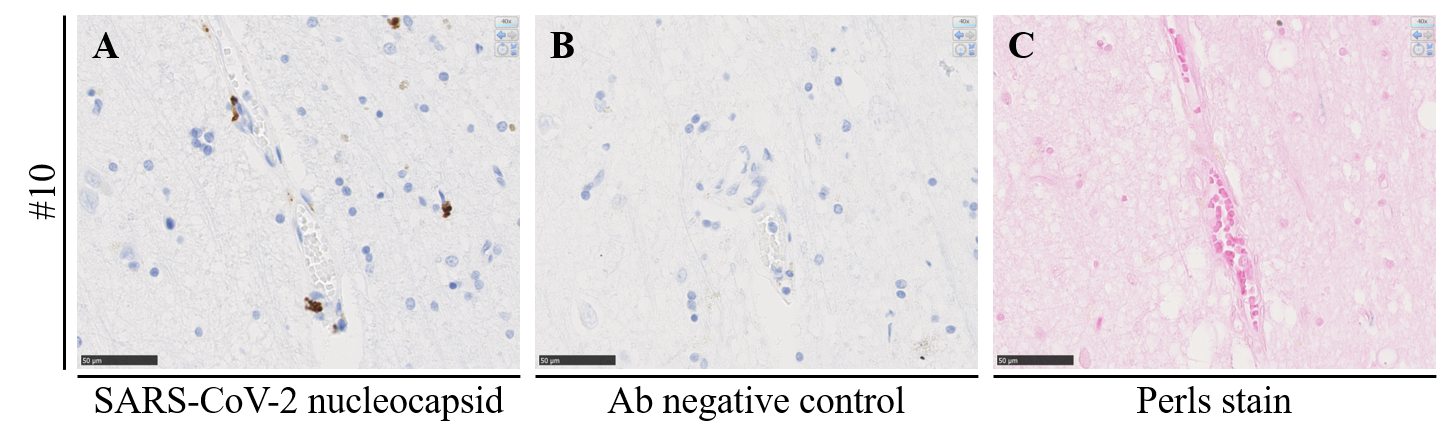

Supplement: Supplementary file 5 — Additional file 5: Fig S1 SARS-CoV-2 immunohistochemistry, negative control and Perls staining of brain sections. Immunohistochemical SARS-CoV-2 nucleocapsid protein, negative control without primary antibody and Perls staining of brain sections from Case 10: viral proteins were detected near some brain capillarieswhile no haemosiderin deposits were observed. No aspecific staining was detected with the secondary antibody only. Scale bars = 50 µm. [file 40478_2023_1566_MOESM5_ESM.tif]
