# Supplementary material for: SARS-Cov-2 infection and neuropathological findings: a report of 18 cases and review of the literature
Source: Acta Neuropathol Commun. 2023 May 10;11:78. doi: 10.1186/s40478-023-01566-1 (PMC10170054; doi:10.1186/s40478-023-01566-1)
Supplement: Supplementary file 4 — Additional file 4: SARS-CoV-2 Immunohistochemistry: Heat-induced epitope retrieval was performed using Dako Target Retrieval Solution pH 6 for 30 min at 97 °C, followed by primary antibody incubation for 20 min at 32 °C and detection with the Dako Envision Flex detection system according to the manufacturer’s protocol. The sections were counterstained with haematoxylin. [file 40478_2023_1566_MOESM4_ESM.docx]

**Supplementary Material 1: SARS-CoV-2 Immunohistochemistry:** Heat-induced epitope retrieval was performed using Dako Target Retrieval Solution pH6 30 min at 97°C, followed by primary antibody incubation 20 min at 32°C and detection with Dako Envision Flex detection system according to the manufacturer’s protocol. The sections were counterstained with hematoxylin.
